# Preparation and characterization of rice husk adsorbents for phenol removal from aqueous systems

**DOI:** 10.1371/journal.pone.0243540

**Published:** 2020-12-04

**Authors:** Samah Babiker Daffalla, Hilmi Mukhtar, Maizatul Shima Shaharun

**Affiliations:** 1 Department of Environment and Agricultural Natural Resources, College of Agricultural and Food Sciences, King Faisal University, Al-Ahsa, Saudi Arabia; 2 Department of Chemical Engineering, Universiti Teknologi PETRONAS, Tronoh, Malaysia; 3 Department of Fundamental and Applied Sciences, Universiti Teknologi PETRONAS, Tronoh, Malaysia; Qatar University, QATAR

## Abstract

Rice husk is a base adsorbent for pollutant removal. It is a cost-effective material and a renewable resource. This study provides the physicochemical characterization of chemically and thermally treated rice husk adsorbents for phenol removal from aqueous solutions. We revealed new functional groups on rice husk adsorbents by Fourier transform infrared spectroscopy, and observed major changes in the pore structure (from macro-mesopores to micro-mesopores) of the developed rice husk adsorbents using scanning electron microscopy. Additionally, we studied their surface area and pore size distribution, and found a greater enhancement of the morphological structure of the thermally treated rice husk compared with that chemically treated. Thermally treated adsorbents presented a higher surface area (24–201 m^2^.g^-1^) than those chemically treated (3.2 m^2^.g^-1^). The thermal and chemical modifications of rice husk resulted in phenol removal efficiencies of 36%–64% and 28%, respectively. Thus, we recommend using thermally treated rice husk as a promising adsorbent for phenol removal from aqueous solutions.

## Introduction

Phenol is a common toxic organic pollutant that is widely present in refineries, petrochemicals, pharmaceuticals, polymeric resins, coal tar distillation, and other industrial processes [1–3]. Phenol is volatile and difficult to degrade in nature, and has acute and chronic effects on human health [1]. The major hazard of phenol is its ability to rapidly penetrate the skin, causing severe burns [1]. The removal of phenol from water before discharge into streams is challenging. Many conventional technologies have been developed to remove phenols from water, including adsorption, chemical oxidation, solvent extraction, membrane processes, and reverse osmosis [2]. Recently, adsorption has become a well-established and powerful technique for phenol removal from aqueous solutions as the appropriate design of the adsorption process has led to high-quality treated discharge [2,4,5]. In general, a good adsorbent is characterized by natural abundance, technical applicability, low cost, lack of toxicity, and large surface area [6].

Rice husk is a promising adsorbent material for removing different contaminants because it is a low-cost and renewable resource. The typical chemical composition of rice husks is about 32% cellulose, 20% hemicellulose, 21% of lignin, and 20% of other organic matter, such as protein and fat [1,7]. Presently, much rice husk is discarded directly into the soil or burnt, which results in environmental pollution. However, thermochemical conversion of rice husk, such as by pyrolysis, gasification, and combustion, can be used to generate value-added by-products [8], and thus substantially reduce the pressure on the environment. Rice husk has been extensively utilized to adsorb phenolic compounds [1,9,10], copper [11–13], lead [13,14], hexavalent chromium [Cr(VI)] [15], malachite green [16], 2, 4-dichlorophenol [17], cadmium [11,14,18,19], zinc and manganese [11,13], selenium [18], humic acids [20], oil and oil products [21], dye [22], and fluoride [23].

Generally, the pretreatment of rice husk can extract soluble organic materials through using various types of modifying agents like base and acid solutions [7]. Rice husk treatment with base solutions have been used to enhance the adsorption properties because the base solutions wash out the inorganic compounds like carbonate and silica from the surface of rice husk [24]. Moreover, burning of rice husk will potentially yield 20% of ash. The produced ash is estimated to contain more than 95% of silica with high porosity as well as large surface area, because it retains the skeleton of cellular structure [25]. In practice, burning temperature and time will lead to producing variable types of ash as these parameters influence the porosity and functional groups of the produced ash. The cellulose–lignin matrix burns away due to combustion, resulting in existence of a porous silica skeleton, which in turns will provide very fine particles having large surface area after grinding [25].

Herein, we report an investigation of thermal and chemical treatments on the sorption ability of rice husk adsorbents in terms of phenol removal. We also characterized and compared the physicochemical properties of all derived adsorbents before and after phenol removal using Fourier transform infrared (FTIR) spectroscopy, and investigated the effect of contact time on the adsorption process using the batch technique.

## Materials and methods

### Chemicals

The chemicals used in this study, such as phenol (99.99% purity), and calcium hydroxide (Ca(OH)_2_) were analytical grade purchased from Merck, Germany. All require solutions were prepared using distilled water.

### Adsorbents preparation

Rice husks were collected from a paddy field belongs to FELCRA Paddy Seed Company (Pusat Benih Padi Felcra Berhad), in Perak state, Malaysia. Normally, the rice samples are provided freely for the academic purposes, as a company tended to collaborate with academic institutions for research development. They were washed multiple times in distilled water to remove soluble impurities and then dried in an oven at 105°C for 24 h. The dried rice husks were milled and passed through different sieve screens, and particles with sizes between 125 and 250 μm were used to prepare the adsorbents by chemical or thermal treatments. For chemical treatment, the rice husks were mixed with 0.5M calcium hydroxide (Ca(OH)_2_) [26], then washed multiple times with distilled water to remove any excess Ca(OH)_2_, and dried in an oven at 60°C for 24 h. Thermal treatment was carried out by burning rice husk using ceramic crucibles in a Bibby Stuart Furnace at 400°C for 1, 2, 3, or 4 h. The four burning times of 1, 2, 3, or 4 h were selected in order to determine the burning time that develops the optimum adsorption performance. The rice husk-based adsorbents code is presented in Table 1.

**Table 1:** Code for developed adsorbents.

Number	Type of treatment	Adsorbent name	Code
**1**	Chemical	RH+0.5M Ca(OH)_2_	B _Ca(OH)2_
**2**	Thermal	RHA400, 1hr	C_400,1_
**3**		RHA400, 2hrs	C_400,2_
**4**		RHA400, 3hrs	C_400,3_
**5**		RHA400, 4hrs	C_400,4_

### Characterization of adsorbents

We characterized the prepared rice husk adsorbents in terms of their surface area using a surface area and pore-size analyzer (Micromeritics ASAP 2020). The amounts of carbon, hydrogen, nitrogen, and sulfur were analyzed using an elemental analyzer (CHNS-932, VTF-900, LECO). Morphological structure and porosity were recorded using a scanning electron microscope (SEM, model LE01430VP, Germany) and a field emission scanning electron microscope (FESEM, model ZEISS SUPRA 55VP). Functional groups were determined using a Fourier transform infrared spectrophotometer (Shimadzu FTIR- 8400S, maximum resolution of 0.85cm^-1^).

### Adsorption kinetics studies

The adsorption kinetics of phenol by various adsorbents was studied in a batch experiment under continuous stirring at 200 rpm at an ambient temperature of 23 ± 1°C. We used 10 g.L^-1^ of the adsorbent and 100 mg.L^-1^ of the initial phenol solution with a normal pH of 5.58. In order to remove the suspended adsorbents in the sorption assays, suspensions were filtered using Whatman syringe filters. The solution phenol concentrations were analyzed after 0, 2, 5, 10, 15, 20, 30, 40, 50, 60, 80, 100, 120, 240, 360, 600, and 1440 min by an Agilent 1100 Series purification system (HPLC) equipped with a diode array detector (DAD). All the batch kinetic studies were conducted twice and the averages of the values were used in the analysis. Eq (1) was used to calculate the phenol removal efficiency,

$$\%Removal\;Efficiency=\frac{\left(C_{o}-C_{eff}\right)}{C_{o}}\times 100(1)$$ (1)

where C_o_ (mg · mg.L^-1^) is the initial concentration of phenol solution, and C_eff_ (mg.L^-1^) is the concentration of phenol at time t.

## Results

### Surface morphologies of the adsorbents

**Fig 1:**
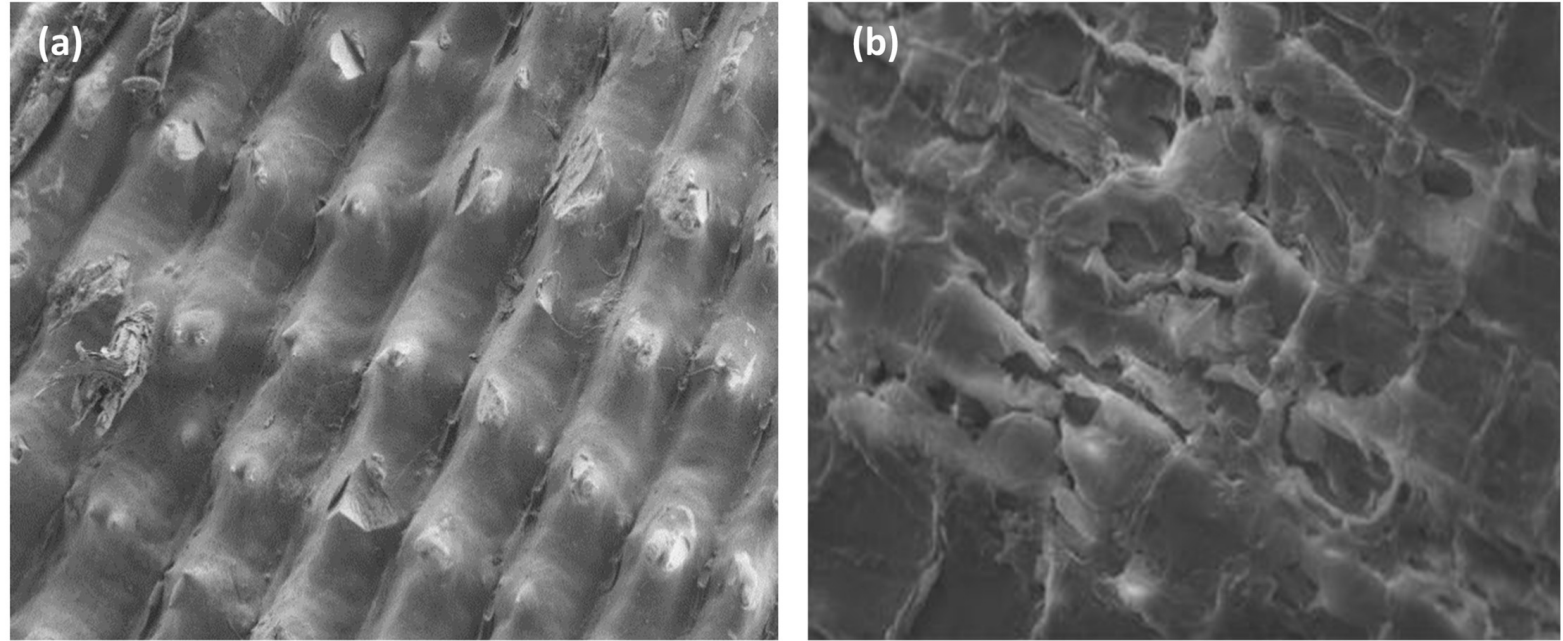
SEM and FESEM for (a) RH, and (b) $B_{\mathrm{Ca}(O{H)}_{2}}$, magnified 1000 times.

The surface adsorbent texture was visualized using SEM and FESEM. Fig 1A shows that the morphological structure of the outer rice husk surface is well organized and corrugated in some places. After the chemical treatment ($B_{\mathrm{Ca}(O{H)}_{2}}$), the surface characteristics were slightly altered (Fig 1B): the surface was degraded, and several rectangular tissues and small holes appeared. Kaur et al. (2020) [27] have reported that pretreatment of rice husks with base and acid removed lignin and hemicellulose, decreased cellulose crystallinity, and eliminated the inorganic materials from the rice husk surface, thus improving its morphology.

**Fig 2:**
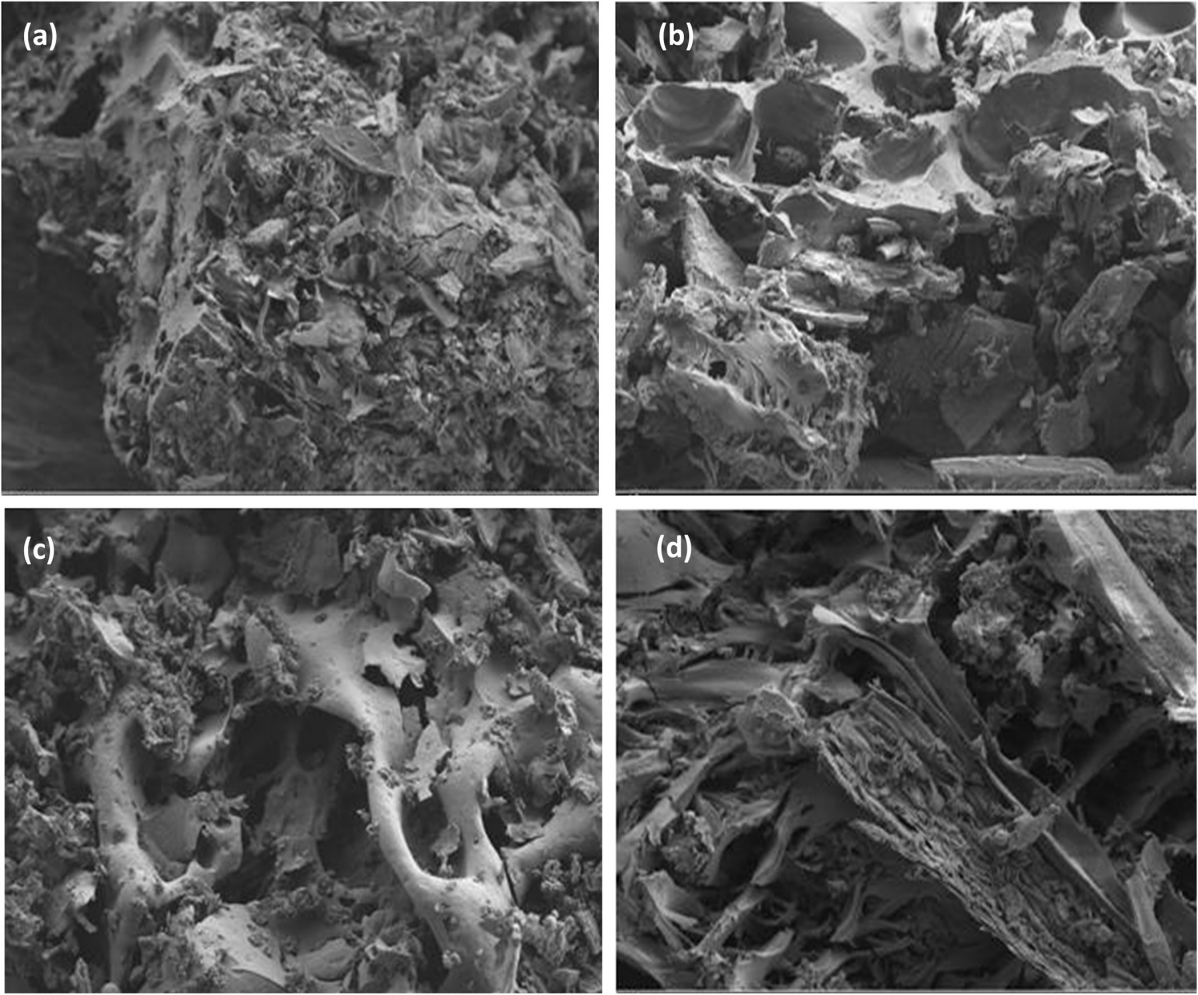
FESEM for adsorbents calcined at temperature 400°C, (a) C_400,1_, (b) C_400,2_, (c) C_400,3_ and (d) C_400,4_ magnified 1000 times.

Fig 2A presents the morphology of the developed rice husk adsorbent after thermal treatment at 400°C for 1 h (C_400,1_). Different pore structures in the thermally treated rice husk (Fig 2A) were not as apparent as in the raw rice husk (Fig 1); the surface structure was destroyed and the cellulose-lignin matrix removed. Pore sizes increased when the burning time was increased from 1 to 2 h (Fig 2B), but then began to collapse as the burning time reached 3 to 4 h (C_400,1_) (Fig 2C and 2D). Such pore size behavior can be explained by the fact that with long burning times, more micropores were converted to large mesopores and macropores.

### Brauneur-Emmett-Teller isotherms and pore size distribution

**Fig 3:**
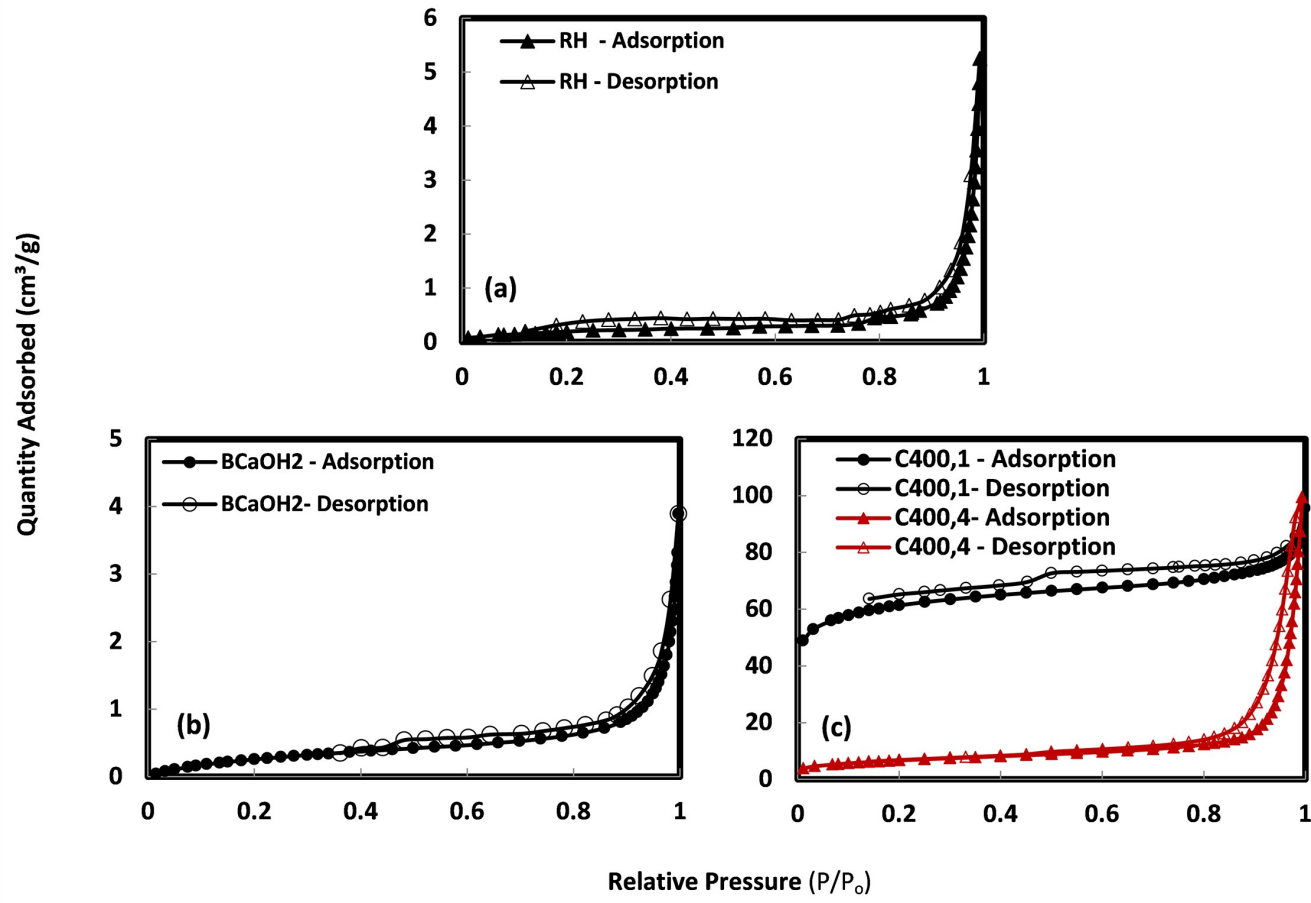
Hysteresis loops of (a) RH, (b) $B_{\mathrm{Ca}(O{H)}_{2}}$, and (c) C_400,1_ and C_400,4_.

The nitrogen adsorption-desorption isotherms of the rice husk adsorbents are illustrated in Fig 3. Fig 3A shows that the rice husk adsorbent has a combination of Types II and IV isotherms as classified by the International Union of Pure and Applied Chemistry, and are characteristic of non-porous or macroporous and mesoporous solids [28,29]. The shapes of the hysteresis slopes of rice husk and $B_{\mathrm{Ca}(O{H)}_{2}}$ are similar (Fig 3A and 3B), with a sharp inflection in the range 0.8–1.0 between P/P_o_ and a hysteresis loop typical of the presence of type H1.

The isotherm of the thermally treated sample (C_400,1_) exhibits a combination of types I and IV (Fig 3C), which are typical for microporous and mesoporous materials, respectively. In contrast, the thermally treated sample (C_400,4_) possesses a combination of isotherms types II and IV, which are characteristic of macroporous and mesoporous solids, respectively (Fig 3C). Therefore, to better understand the structural changes that occur in the transformation from raw rice husk to ashes, we combined the SEM and FESEM analysis with the BET porosity data. As shown in Fig 1A, the surface structure of the rice husk changed from non-porous when untreated to microporous when heated at 400°C for 1 h to form an ash (Fig 2A). The ash surface was also characterized by the lack of star-like shapes. When the burning time was increased to 4 h, the ash started to produce a mesoporous adsorbent (Fig 2D), because many micropores collapsed or merged into mesopores.

Table 2 presents the BET surface area, pore volume, pore diameter, and microporosity of the adsorbents. It can be seen that the BET surface area of sample $B_{\mathrm{Ca}(O{H)}_{2}}$ was 55% less than that of the raw rice husk. The total pore volume of the base-treated sample was higher than that of the raw rice husk, constituting 0.0058 and 0.0055 cm^3^.g^-1^, respectively. The average pore diameter of the base-treated rice husk was significantly higher than that of the raw rice husk, being 16.2 and 3.1 nm, respectively. Such changes in the rice husk structure can be explained by the activating agents (base) causing the original rice husks to decrease in size by micropores being partially blocked and partially rearranged to macropores and mesopores. As such, the increased percentage of macropores and mesopores and the decreased percentage of micropores would lead to a larger average rice husk pore diameter. We attributed the surface area decrease of the Ca(OH)_2_-treated adsorbent to pore opening. For thermally treated samples, the BET surface area of the ash increased from 7.1 m^2^.g^-1^ (raw rice husks) to 201 m^2^.g^-1^ (C_400,1_) and the decreased to 24.0 m^2^.g^-1^ (C_400,4_). The pore volume increased significantly from 0.055 cm^3^.g^-1^ (raw rice husks) to 0.128 cm^3^.g^-1^ (C_400,1_), while the total pore diameter of the rice husk slightly decreased from 3.09 (raw rice husks) to 2.55 nm (C_400,1_) (Table 2). As the heating time increased to 4 h, the total pore diameter increased to 3.53 nm (C_400,4_).

**Table 2:** Pore textural characteristics of adsorbents.

Adsorbent	BET surface area (m^2^.g^-1^)	Micropore area (m^2^.g^-1^)^a^	Micropore volume (cm^3^.g^-1^)^a^	Total pore volume (cm^3^.g^-1^)^b^	Average pore diameter (nm)^c^	Microporosity (%)^d^
**RH**	7.14	1.44	0.00	0.0055	3.09	0.00
$B_{\mathrm{CaO}H_{2}}$	3.19	0.00	0.00	0.0058	16.24	0.00
**C**_**400,1**_	201	125	0.06	0.13	2.55	48.0
**C**_**400,4**_	24.0	1.46	0.0005	0.12	3.53	0.41

^a^Applying Barrett-Joyner-Halenda (BJH) model

^b^Single point adsorption total pore volume

^c^Adsorption average pore width (4V/A by BET)

^d^Microporosity = (Micropore volume/Total pore volume) × 100%.

**Fig 4:**
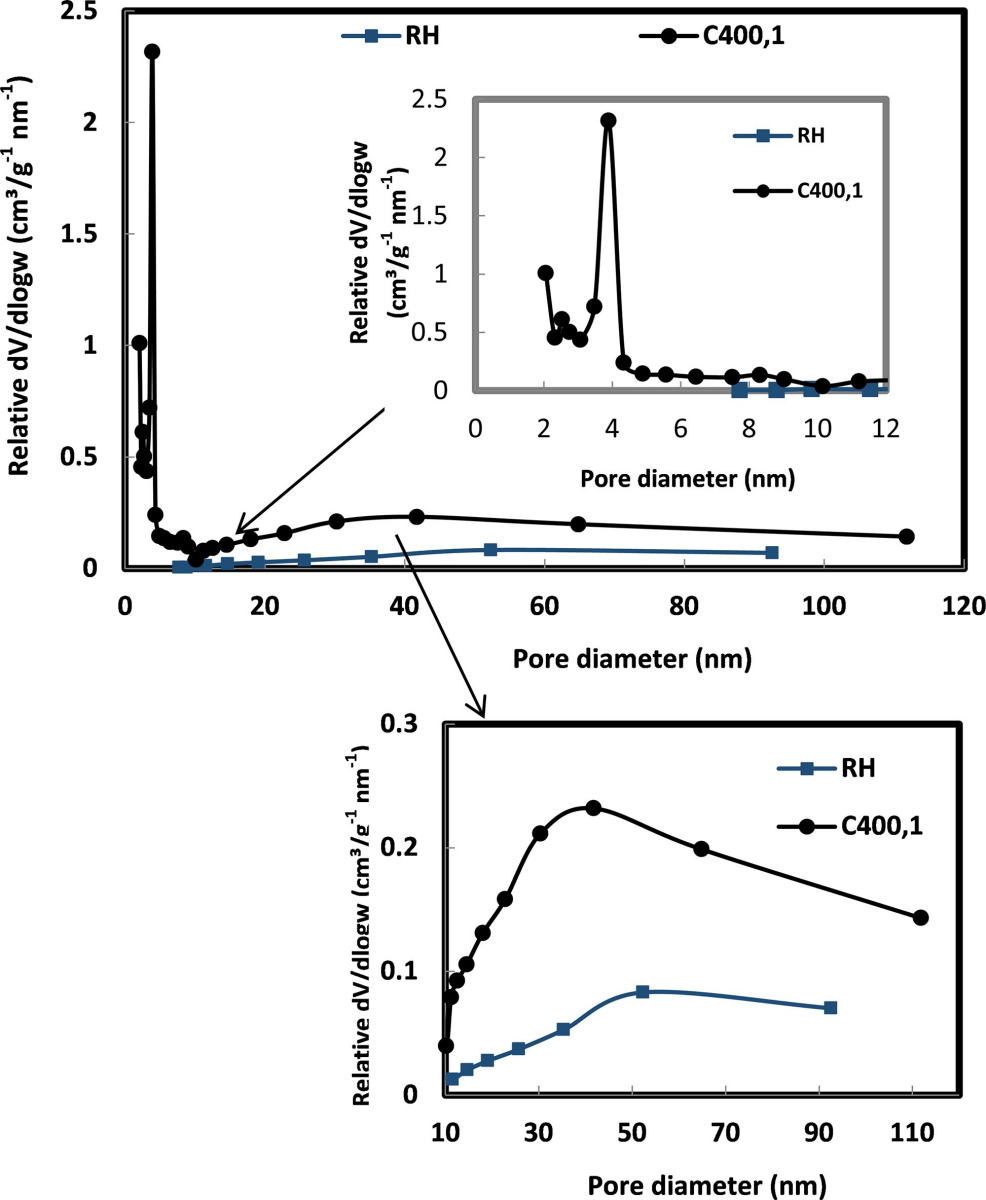
Pore size distribution of RH, and C_400,1_.

We conclude that the rice husk treated with calcium hydroxide ($B_{\mathrm{Ca}(O{H)}_{2}}$) has qualities of a macroporous adsorbent, defined by a low surface area (3.19 m^2^.g^-1^) and negligible pore volume (0.0058 cm^3^.g^-1^), whereas the thermally treated sample burned at 400°C for 1 hour (C_400,1_) is a micro-mesoporous adsorbent with a high surface area (201 m^2^.g^-1^) and higher pore volume (0.13 cm^3^.g^-1^). The pore size distributions of the raw rice husk and C_400,1_ samples are presented in Fig 4. The raw rice husk shows a broad pore distribution centered at approximately 52.2 nm, while for sample C_400,1_, the pore size distribution shifts towards the micropore region centered at 2 nm and the mesopore region centered between (2.7–3.9 nm) and 40 nm.

### Elemental analysis

Table 3 lists the elemental analysis results for the developed rice husk adsorbents, where the main elements in the raw material were carbon and silicon. Table 3 also shows that for the chemically treated rice husk ($B_{\mathrm{Ca}(O{H)}_{2}}$), there was a slight decrease in carbon, hydrogen, nitrogen, and sulfur contents, and that the silicon content was minimal. Such a decrease in these elements was likely due to reaction of the activating agent with rice husk, which resulted in dissolution of the inorganic material on the surface of the raw rice husk [26]. For the ash samples (C_400,1_–C_400,4_), the carbon content decreased with increasing burning time: from 41.2% (raw rice husk) to 40.9% (C_400,1_) and to 3.1% (C_400,4_). The silicon content decreased from 12.0% (raw rice husk) to 4.1% (C_400,1_), but then increased to 27.8% when the burning time was increased to 4 h (C_400,4_). This is due to the reaction for the rice husk proceeded with the two stages. The first stage showed rapid mass decrease caused by cellulose decomposition at the first hour. Then, at the second stage, lignin decomposed for pyrolysis and its char burned for combustion [27].

**Table 3:** Elemental analysis of adsorbents.

Treatment method	Adsorbent	Carbon^a^ (wt.%)	Hydrogen^a^(wt.%)	Nitrogen^a^ (wt.%)	Sulphur^a^ (wt.%)	Silicon^b^ (wt.%)
**Raw material**	RH	41.2	6.1	1.1	0.06	12
**Base**	$B_{\mathrm{CaO}H_{2}}$	37	5.7	0.97	0.04	1.2
**Thermal at 400°C**	C_400,1_	40.9	2.2	2.26	0.1	4.1
	C_400,2_	24.7	1.7	2.25	0.14	8.6
	C_400,3_	7	1.3	1.66	0.15	24.2
	C_400,4_	3.1	0.8	0.98	0.16	27.8

^a^ CHNS Analyzer;

^b^ Energy-Dispersive X-Ray Spectroscopy (EDX).

### Analysis of surface functional groups

**Fig 5:**
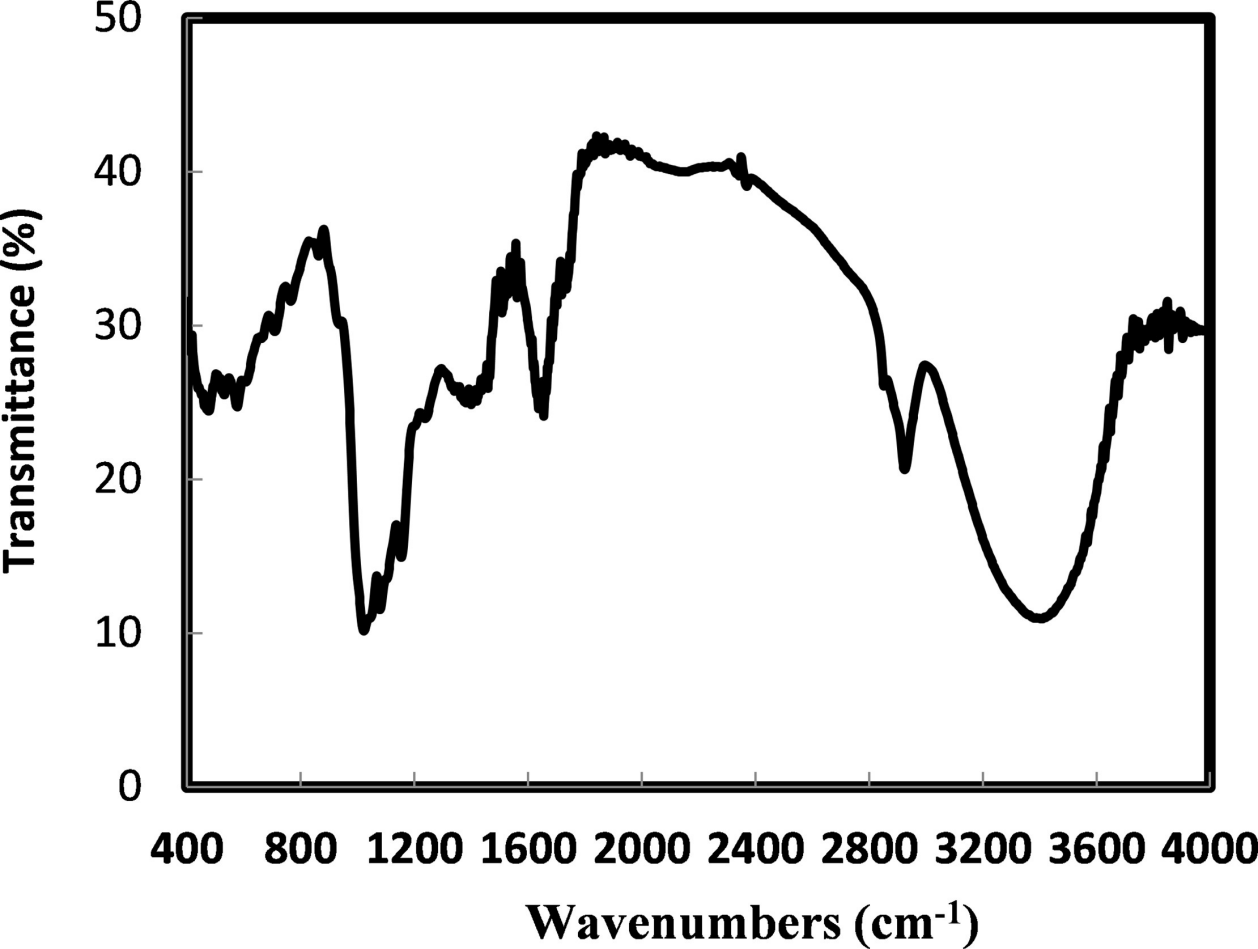
FTIR spectra of RH.

**Fig 6:**
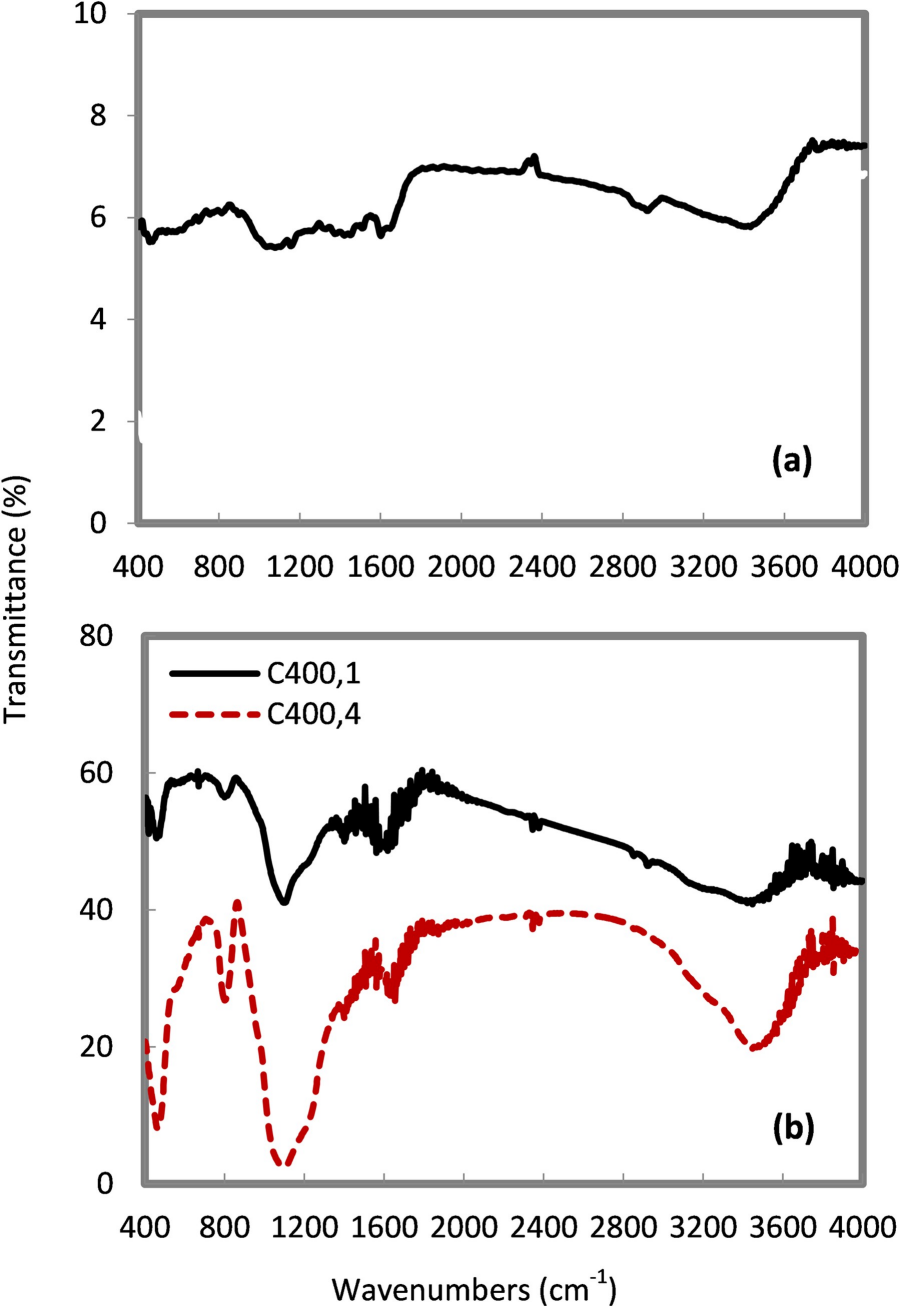
FTIR spectra of (a) $B_{\mathrm{Ca}(O{H)}_{2}}$, and (b) C_400,1_ and C_400,4_.

The FTIR spectra of the tested adsorbents are shown in Figs 5 and 6. In Fig 5, the FTIR spectra of the raw rice husk shows peaks around 3404.3 cm^-1^ (-O-H groups), 2925.8 cm^-1^ (C-H groups), 1641.3–1737.7 cm^-1^ (C = O group), 1546.8–1652.9 cm^-1^ (C = C groups), 1461.9 cm^-1^ (CH_2_ and CH_3_ groups), 1380 cm^-1^ (CH_3_ group), 1379.0 cm^-1^ (aromatic CH stretching and carboxyl-carbonate structures), 1153.4–1300 cm^-1^ (CO group), 1238 cm^-1^(CHOH group), 1080 cm^-1^ (Si-O-Si group), and 862.1–476.4 cm^-1^ (Si-H group) [28,30]. In the chemically treated sample (Fig 6A), the peak around 1750–1725 cm^-1^ disappeared as the base treatment converted the carboxylic ester group to the carboxylate and alcohol groups. Moreover, the silica functional group (Si-O-Si) of the chemically treated sample appeared less intense than that of the raw rice husk due to the removal of inorganic materials from the rice husk surface by the base treatment [24]. This observation was confirmed by the negligible silicon content of the chemically treated sample (Table 3) after the base treatment.

Burning rice husk at 400°C (Fig 6B) resulted in a loss of the C-H, C-C, C-O, C-O-C, and C-O-H bands, and the spectra were dominated by the primary functional groups of C = O and silica functional groups of Si-O-Si, Si-H, and Si-OH [31]. These results show that the functional groups in the rice husk adsorbents are dependent on the treatment method: chemically treated samples are characterized by the OH, Si-O-Si, and Si-H functional groups, whereas thermally treated samples possess C-C, C-O and C-O-C, C-O-H, C = O, Si-O-Si, and Si-H functional groups. The following section describes the functional groups which participated in phenol adsorption.

### Effect of surface functional groups on phenol adsorption

Table 4 shows how the FTIR peaks shifted after the phenol adsorption. Some of the FTIR peaks pertaining to $B_{\mathrm{Ca}(O{H)}_{2}}$, and C_400,1_ shifted to either lower or higher wavenumbers, and it was concluded that various silicon (Si-OH, Si-O-Si, and -Si-H) and carbon (C-H, C = C, C≡C, CO, and C = O) functional groups contributed to the adsorption of phenol onto the adsorbent surface in both chemically and thermally treated samples.

**Table 4:** FTIR spectra of adsorbents before and after phenol sorption (I, before phenol sorption; II, after phenol sorption).

Adsorbent	Functional group					
$B_{\mathrm{CaO}H_{2}}$	Si-OH, -OH	C-H	C≡C	CO	Si-O-Si	Si-H
**I**	3414	2922	2303	1204	1041	897
**II**	3404	2930	2338	1209	1055	856
**C**_**400,1**_	Si-OH,-OH	C = C	C = O	Si-O-Si	Si-H	
**I**	3425	1601	1659	1099	679	
**II**	3383	1612	1655	1094	678	

### Kinetic adsorption studies

**Fig 7:**
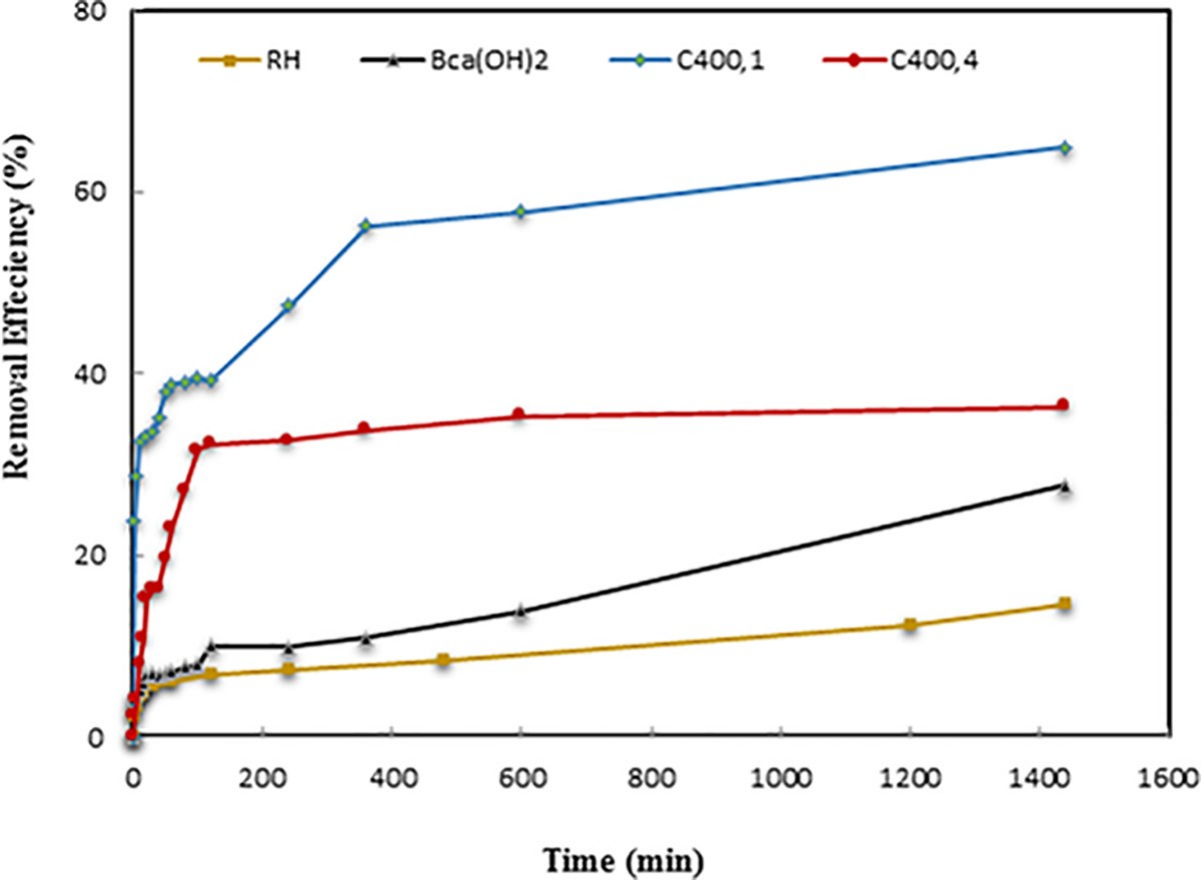
Effect of contact time on uptake of phenol on RH, $B_{\mathrm{Ca}(O{H)}_{2}}$, C_400,1_ and C_400,4_ (initial concentration, 100 mg.L^-1^; adsorbent dose, 10 g.L^-1^; pH 5.6; 23±1°C).

Adsorption kinetic experiments were performed by equilibrating adsorbents with 100 mg.L^-1^ of phenol solution at pH 5.6 and 10 g.L^-1^ of the adsorbent. A statistical analyses for RH, $B_{\mathrm{Ca}(O{H)}_{2}}$, C_400,1_, and C_400,4_ was conducted (Table 5) in order to assess the extent to which the resultant data is concise, where standard errors were found to be small in value. Adsorption kinetic curves revealed that the adsorption of phenol on RH, $B_{\mathrm{Ca}(O{H)}_{2}}$, C_400,1_, and C_400,4_ occurred in two stages: a rapid initial adsorption followed by a long period of much slower phenol uptake until equilibrium was reached (Fig 7). Fig 7 shows that the RH and chemically treated sample ($B_{\mathrm{Ca}(O{H)}_{2}}$) had a low removal efficiencies of 14.6% and 27.8%, respectively, likely due to their low surface areas and high pore diameters (Table 2). Moreover, the $B_{\mathrm{Ca}(O{H)}_{2}}$ adsorbent was characterized as a non-porous material (Fig 3B), and thus is unfavorable for adsorption of phenol.

**Table 5:** Descriptive statistics of adsorbents removal efficiency.

Adsorbent		RH	$B_{\mathrm{Ca}(O{H)}_{2}}$	C_400,1_	C_400,4_
**Number of samples**		12	16	16	16
**Removal Efficiency (%)**	**Minimum**	2.3	4.1	23.8	2.3
	**Maximum**	14.6	27.8	64.9	36.4
	**Mean**	5.9	7.0	21.4	38.4
	**Std. Deviation**	3.4	5.4	11.1	11.2
	**Std. Error**	1.0	1.4	2.8	2.8

Fig 7 shows the temporal changes in phenol removal efficiency of the thermally treated samples (C_400,1_ and C_400,4_). The adsorption behavior of the thermally treated samples was similar to that of the chemically treated sample (Fig 7), but the phenol removal efficiency was significantly affected by their surface areas and pore diameters. As shown in Fig 7, the rice husk ashes were characterized by high phenol removal efficiency of 36%–65% as a result of favorable microporosity and mesoporosity for phenol adsorption.

Fig 7 also shows that the phenol removal efficiency of the rice husk ashes decreased with increasing the burning time, and is consistent with the data on surface area (Table 2) and morphological structure (Fig 2). As the burning time increased to 4 h, the surface area and microporosity decreased, while the pore diameter increased, with pores collapsing (Table 2), and as a result, we suggest that the optimum time for burning rice husks at 400°C is 1 h (Fig 2).

## Discussion

After characterizing the rice husk adsorbents in terms of their surface area, pore size distribution, morphology, and functional groups, we showed that they possessed different characteristics depending on the treatment method. Other researchers have reported similar results [6,18,32,33], and stated that rice husk pretreatment enhances the phenol removal efficiency and adsorption capacity.

Chemical treatment revealed that the surface layers of rice husk tissues were removed, whereas thermal treatment at a temperature of 400°C created undulating surfaces in the rice husk with small and large pores. We also noted that thermal treatment enhanced the rice husk morphology better than the chemical treatment. The surface area of the thermally treated samples was significantly higher than that of the chemically treated sample, and this result is similar to that reported by Akhtar et al. (2006) [17]. The rice husk porosity was increased by both chemical or thermal pretreatments as a result of extraction of lignin and hemicellulose as well as reduction of cellulose crystallinity, and the increased number of pores led to better phenol removal. As porosity describes the number of pores present in a sample, it also enhances the adsorption capacity of the adsorbent, in agreement with other findings [7]. We compared the BET surface area of the rice husks with and without pretreatment (Table 2), and observed that the BET surface area and total pore volume decreased with increasing burning time, whereas the pore diameter increased. We suggest that such behavior is due to the collapse of pores, as confirmed by the morphology of the adsorbents (Fig 2). The surface area of the ashes studied herein was higher than that published by Kaur et al. (2020) [28], Mandal et al. (2019) [34] and Srivastava et al. (2006) [30], who burned rice husk at 500°C for 30 min, rice husk at 600°C for 4 h, and bagasse and obtained surface areas of 144.23 m^2^.g-^1^, 57.5 m^2^.g-^1^, and 168.4 m^2^.g-^1^, respectively.

According to the elemental analysis, an increase in the burning time significantly decreased the amount of carbon and increased the weight percent of silicon in the thermally treated samples (Table 2), and this result is similar to the report of Zou and Yang (2019) [32].

We also report that the rice husk adsorbents possess different functional groups depending on the treatment method used, and these may be shifted to lower or higher wavenumbers as a result of phenol adsorption (Table 4). These spectral shifts confirm that rice husk is a good quality adsorbent for phenol removal from water, and correlate well with other reported studies [1,2].

The C_400,1_ adsorbent (Fig 7B) had a higher phenol removal efficiency than the $B_{\mathrm{Ca}(O{H)}_{2}}$ adsorbent (Fig 7A), which may be explained by its higher surface area, microporosity, and pore diameter (Table 2). Thus both the adsorbent pore size and the adsorbate molecule size have to be considered when explaining the adsorption process, and the micropore structure of C_400,1_ is more favorable for the adsorption of phenol. Which has a molecular diameter of approximately 0.66 nm, Su et al. (2005) [35] have also reported that phenol removal can be mainly explained by micropore filling, although we show here that the surface functional groups also significantly affects phenol adsorption. The phenol removal efficiency (65%) studied herein was found to be higher than those reported for various types of adsorbents, which were of 29.6% [36], 60% [37] and 61% [38] for rice straw ash, natural clay, and aspergillus versicolor, respectively.

## Conclusions

In this study, we developed different rice husk adsorbents for phenol removal, and systematically characterized their morphology, surface area, elemental composition, and functional groups. We found that the physicochemical properties of the adsorbents were dependent on the treatment method used. Thermally-treated samples had a higher surface area (24–201m^2^.g^-1^) than a chemically treated sample (3.2 m^2^.g^-1^), and FTIR analysis confirmed that certain functional groups (e.g., -OH, C-C, C = C, C≡C, C-O, C = O, Si-OH, Si-O-Si, and -Si-H) contributed to phenol adsorption on the adsorbent surfaces. In addition, thermally treated samples showed better phenol removal efficiency (36.4%–64.9%) than the chemically treated sample (27.8%). However, the thermally-treated rice husk-derived materials (e.g., C_400,1_, C_400,4_) varied in their efficiency for phenol removal dependent on their physiochemical properties. Overall, this study has demonstrated that rice husk is a renewable natural material that can be thermally treated to produce an adsorbent with great potential for phenol removal from aqueous solutions.

## Supporting information

S1 Fig FTIR spectra of C_400,2_. (DOCX)

S2 Fig FTIR spectra of C_400,3_. (DOCX)
