# Supplementary material for: Preparation and characterization of rice husk adsorbents for phenol removal from aqueous systems
Source: PLoS One. 2020 Dec 4;15(12):e0243540. doi: 10.1371/journal.pone.0243540 (PMC7717560; doi:10.1371/journal.pone.0243540)
Supplement: S1 Fig — (DOCX) [file pone.0243540.s001.docx]

**Figure S-1:** FTIR spectra of C_400,2_
